# Long Noncoding RNA and mRNA Expression Profiles in the Thyroid Gland of Two Phenotypically Extreme Pig Breeds Using Ribo-Zero RNA Sequencing

**DOI:** 10.3390/genes7070034

**Published:** 2016-07-09

**Authors:** Yifei Shen, Haiguang Mao, Minjie Huang, Lixing Chen, Jiucheng Chen, Zhaowei Cai, Ying Wang, Ningying Xu

**Affiliations:** 1 College of Animal Science, Zhejiang University, Hangzhou 310058, China; 06dwkxsyf@zju.edu.cn (Y.S.); 11517001@zju.edu.cn (H.M.); 11417014@zju.edu.cn (M.H.); 21417001@zju.edu.cn (L.C.); 11217007@zju.edu.cn (J.C.); 2 Laboratory of Animal Research Center, Zhenjiang Chinese Medical University, Hangzhou 310053, China; czw1234@163.com; 3 College of Mechanics, Taiyuan University of Technology, Taiyuan 030024, China; Wangying03@tyut.edu.cn

**Keywords:** Ribo-zero RNA sequencing, lncRNA, thyroid, pig

## Abstract

The thyroid gland is an important endocrine organ modulating development, growth, and metabolism, mainly by controlling the synthesis and secretion of thyroid hormones (THs). However, little is known about the pig thyroid transcriptome. Long non-coding RNAs (lncRNAs) regulate gene expression and play critical roles in many cellular processes. Yorkshire pigs have a higher growth rate but lower fat deposition than that of Jinhua pigs, and thus, these species are ideal models for studying growth and lipid metabolism. This study revealed higher levels of THs in the serum of Yorkshire pigs than in the serum of Jinhua pigs. By using Ribo-zero RNA sequencing—which can capture both polyA and non-polyA transcripts—the thyroid transcriptome of both breeds were analyzed and 22,435 known mRNAs were found to be expressed in the pig thyroid. In addition, 1189 novel mRNAs and 1018 candidate lncRNA transcripts were detected. Multiple TH-synthesis-related genes were identified among the 455 differentially-expressed known mRNAs, 37 novel mRNAs, and 52 lncRNA transcripts. Bioinformatics analysis revealed that differentially-expressed genes were enriched in the microtubule-based process, which contributes to THs secretion. Moreover, integrating analysis predicted 13 potential lncRNA-mRNA gene pairs. These data expanded the repertoire of porcine lncRNAs and mRNAs and contribute to understanding the possible molecular mechanisms involved in animal growth and lipid metabolism.

## 1. Introduction

The thyroid is one of the largest endocrine glands in the body, mainly synthesizing and secreting two vital thyroid hormones (THs), tetraiodothyronine or thyroxine (T4) and triiodothyronine (T3). THs play critical roles in differentiation, growth, metabolism, and reproduction [1]. Increased levels of THs can stimulate almost all aspects of carbohydrate metabolism, increase the basal metabolic rate, and stimulate both lipogenesis and lipolysis—although lipolysis is more influenced than synthesis [2,3]. Thyrocytes—the cells responsible for producing and secreting T4 and T3—integrate complicated extracellular signals from the hypothalamus (thyrotropin releasing hormone, TRH) and the pituitary (thyroid stimulating hormone, TSH), the feedback from serum THs [4], and from other factors, such as iodine and leptin [5,6], which then influence the intracellular signaling pathways modulating THs synthesis and secretion. Thus, many interacting genes determine the regulation of THs synthesis and secretion. Among others, the genes encoding thyroglobulin (*Tg*), thyroid stimulating hormone receptor (*TSHR*), solute carrier family 5 member 5 (*SLC5A5*), iodotyrosine deiodinase (*IYD*), and dual oxidase 2 (*DUOX2*), as well as the calcium signaling pathway, glutathione metabolism, lysosome, and tyrosine metabolism signaling pathways, are involved in the synthesis and secretion of THs [7].

Long noncoding RNAs (lncRNAs) are a type of RNA transcript, longer than 200 bases but without evident protein-coding capacity. Accumulating evidence suggests that lncRNAs regulate gene expression during cellular development and homeostasis by recruiting epigenetic complexes or directly affecting the process of transcription [8,9,10]. The expression of lncRNAs is highly regulated, displaying discrete temporal and spatial expression patterns [11,12], and some groups have revealed their crucial role in the normal development and function of several endocrine organs, such as the pancreas, prostate gland, and adipose tissue [13]. However, little is known about the physiological function of lncRNAs in the thyroid gland.

Recent studies suggest that non-polyadenylated (polyA) RNA transcripts (both protein-coding and noncoding) are functionally important [14,15]. Although high-throughput RNA sequencing (RNA-seq) elucidates on global gene expression profiles, the standard method of enriching polyA RNAs with oligo (dT) primers cannot be used to reveal non-polyA transcripts and partially degraded mRNAs. This limitation has been overcome by the Ribo-Zero RNA-seq, a new method that can capture both polyA and non-polyA transcripts [16].

The pig (*Sus scrofa*) is an economically important food source, corresponding to approximately 40% of all meat consumed worldwide. The Jinhua pig is a Chinese indigenous fat-type pig breed that has low growth rate and body weight, but high fat deposition. The Yorkshire pig, on the contrary, is a lean-type pig breed characterized by high growth rate and body weight, but low fat deposition. Therefore, Jinhua and Yorkshire pigs are ideal models for studying animal growth and fat deposition.

In this study, we compared the transcriptome profiles of the thyroid gland of Jinhua and Yorkshire pigs using Ribo-Zero RNA-seq. To our knowledge, this is the first description of lncRNA expression profiles in the porcine thyroid. This study aimed to extend the transcript catalog (both mRNAs and lncRNAs) in pig species, identifying putative mRNAs, lncRNAs, and pathways that are involved in the regulation of animal growth and fat deposition.

## 2. Materials and Methods

### 2.1. Animal Material

All procedures described here were conducted under an experimental license from the Animal Care and Use Committee of Zhejiang University, in agreement with the relevant guidelines and regulations imposed by the Administration of Affairs Concerning Experimental Animals. Three castrated Jinhua pigs and three castrated Yorkshire pigs aged 120 days were humanely killed and their body weights were recorded. Thyroid glands were weighted and thyroid weight indices were calculated as thyroid weight/ body weight ratios (g/kg). Thyroid glands were cut up and preserved in liquid nitrogen within 10 min post-slaughter, and then stored at −80 °C until RNA extraction.

### 2.2. Serum THs Assays

Blood samples were collected from the anterior vena cava of each animal, kept at 37 °C for 2 h, and then centrifuged at 4 °C for 10 min (3000 × *g*). Serum was collected and its total thyroxine (TT4), total triiodothyronine (TT3), free thyroxine (FT4), and free triiodothyronine (FT3) concentrations were measured through an enzyme-linked immunosorbent assay (ELISA) provided at the Affiliated Hospital of Hangzhou Normal University.

### 2.3. RNA Isolation, Library Preparation, and Sequencing

Total RNA was isolated from each individual sample using TRIzol™ reagent (Invitrogen, Carlsbad, CA, USA), according to the manufacturer’s instructions. RNA was quantified using a Qubit RNA Assay Kit in a Qubit 2.0 Fluorometer (Life Technologies, Carlsbad, CA, USA), and checked for purity and integrity in a Bioanalyzer 2100 system (Agilent Technologies, Palo Alto, CA, USA). RNA integrity number (RIN) was larger than 8.0 in all samples.

Approximately 3 μg RNA per sample (two individuals per breed) were used to construct the complementary (cDNA) library, according to the following procedures: ribosomal RNA (rRNA) was removed from total RNA by Epicentre Ribo-zero™ rRNA Removal Kit (Epicentre, Madison, WI, USA); rRNA-depleted RNA was then used to generate strand-specific RNA-seq libraries using the NEBNext^®^ Ultra™ Directional RNA Library Prep Kit for Illumina^®^ (NEB, Ipswich, UK), following manufacturer’s recommendations. Briefly, after RNA fragmentation, double-stranded cDNA was synthesized replacing dTTPs with dUTPs in the reaction buffer used in second strand cDNA synthesis. The resulting double-stranded cDNA was ligated to NEBNext^®^ adaptors, after being end-repaired and A-tailed, and 150–200 bp cDNA fragments were isolated. Single strand cDNA was then obtained using USER Enzyme. Finally, PCR amplification was performed to enrich cDNA libraries.

Sequencing was performed on an Illumina^®^ Hiseq 2500 (Illumina, San Diego, CA, USA) instrument using the TruSeq PE Cluster Kit v3-cBot-HS (Illumina^®^) to generate 125 bp paired-end reads.

### 2.4. Mapping to the Reference Genome

Quality control and reads statistics were determined by FASTQC v0.11.2 [17]. The Phred score (Q20, Q30) and GC content of the raw reads were calculated. After discarding the reads containing adapter, reads containing over 10% poly-Ns, and reads of low quality (>50% of bases with Phred scores <5), the remaining clean reads were aligned to the reference pig genome (*S. scrofa* 10.2) using TopHat v2.0.9 [18] and its underlying aligner Bowtie v2.0.6 [19]. TopHat run parameters were set to default except for “-library-type fr-firststrand”. The number of reads annotated in mRNAs, misc_RNAs, ncRNAs, precursor RNAs, pseudogenes, rRNAs, and tRNAs were counted in HTSeq v0.5.4p5 [20]. 

### 2.5. Transcriptome Assembly

Mapped reads belonging to each sample were assembled in Scripture (beta2) [21] using the default parameters, and in Cufflinks v2.1.1 [22] using the default parameters, except for “min-frags-per-transfrag = 0” and “-library-type fr-firststrand”. Transcripts resulting from the two assemblers were pooled, and they were considered reliable if it found for at least two samples or in both assemblers.

### 2.6. Identification of lncRNA and Novel mRNA

Assembled transcripts were processed as follows:

1. The reads coverage of every transcript was calculated by Cufflinks, and those with reads coverage less than three were eliminated;
2. Single exon transcripts and transcripts <200 bp were excluded;
3. Transcripts blasted to known mRNA and belonged to pseudogenes, pre-microRNA, tRNA, rRNA, and snoRNA were removed;
4. CNCI v2 (score < 0) [23], CPC-0.9-r2 (score < 0) [24], Pfam-scan v1.3 (E-value < 0.001) [25], and PhyloCSF v20121028 (no sufficiently long ORFs found) [26] were used to evaluate the coding potential of transcripts. Transcripts revealing coding potential in any of the four tools were filtered out.

Transcripts that passed all the filters mentioned above were considered candidate lncRNAs, and those with coding potential were considered novel mRNAs. Candidate lncRNAs were blasted to pig lncRNAs in the ALDB v1.0 database.

### 2.7. Homology Analysis with lncRNAs in Human and Mouse

Human and mouse lncRNAs were downloaded from NONCODE v5 database [27], which included several public databases (Ensembl [28], RefSeq [29], lncRNAdb [30], and GENCODE [31]). Blastn was used to search for homology between candidate lncRNAs identified in our study and human or mouse lncRNAs in the NONCODE v5 database, considering a 1 × 10^−6^ E-value threshold.

### 2.8. Differential Expression Analysis

The expression levels of mRNAs and lncRNAs were calculated in fragments per kilo-base of exon per million mapped fragments (FPKM) using the Cuffdiff tool in Cufflinks v2.1.1 [22]. In addition, Cuffdiff was applied to determine differentially-expressed mRNAs and lncRNAs using a model based on the negative binomial distribution. A *q*-value < 0.05 (Benjamini and Hochberg’s false discovery rate) was set as the threshold for significantly differential expression. Hierarchical clustering was performed to visualize mRNAs and lncRNAs differential expression patterns among samples.

### 2.9. Functional Enrichment Analysis

Gene Ontology (GO) analysis of the differentially-expressed mRNAs was performed using the GOseq R package (Release2.12) [32], considering GO terms with corrected *p*-value < 0.05 as significantly enriched. Pathway analysis, using KO-Based Annotation System (KOBAS) v2.0 [33], allowed statistical evaluation of the differentially-expressed mRNAs in KEGG pathways.

### 2.10. Target Gene Prediction

Differentially-expressed lncRNAs were selected for target prediction. The cis role of lncRNAs was their acting on neighboring target genes [34]. In this study, mRNAs 100 kb up- and down-stream of the differentially-expressed lncRNAs were evaluated, as they might be regulated by the adjacent lncRNAs. In order to reduce false positives, the differentially-expressed mRNAs were selected as potential target genes.

### 2.11. Validation of the Sequencing Data by qRT-PCR

Quantitative real-time PCR was performed on an ABI Step One Plus system (Applied Biosystem, Carlsbad, CA, USA) using SYBR Premix Ex Taq kit (TaKaRa, Dalian, China) with specific primers (Table S11). Gene relative-expression levels were quantified based on GAPDH gene expression by using ΔΔCt method [35]. Three independent biological replicates were used. All measurements were performed in triplicate. Statistical differences between two pig breeds were evaluated using Student’s *t*-test and *p* < 0.05 as the threshold for significance.

## 3. Results

### 3.1. Body Index and THs Concentrations in the Serum of the Two Porcine Breeds

As shown in Table 1, body weight and thyroid gland weight of Yorkshire pigs were significantly higher than in Jinhua pigs (*p* < 0.05); however, the thyroid index was almost identical for the two pig breeds. Yorkshire pigs had significantly higher levels of TT4 and TT3 than Jinhua pigs (*p* < 0.05), and higher levels of FT4 and FT3 than Jinhua pigs, although these were not statistically significant (*p* > 0.05).

### 3.2. Characterization of the Thyroid Gland Transcriptome

The four Ribo-zero RNA sequencing libraries (two Jinhua and two Yorkshire) were sequenced on the Illumina^®^ HiSeq 2500 platform. Sequencing yielded about 351 million 125-bp paired-end reads. Approximately 83% of these reads were mapped to the pig reference genome (*S. scrofa* 10.2) using Tophat, and about 72% aligned with unique loci (Table 2). HTSeq was used to calculate the proportion of reads aligning to mRNAs, misc_RNAs, ncRNAs, precursor RNAs, pseudogenes, rRNAs, and tRNAs (Table S1). Most reads were matched mRNAs (51%–58%). However, 33%–39% of the reads were mapped outside of annotated loci.

### 3.3. Known mRNA Profiling in Pig Thyroid

There are 38,222 known mRNAs so far annotated in the pig genome, according to the RefSeq database. 22,435 mRNAs were expressed in the pig thyroid, which represents ~59% of the total annotated pig mRNAs (Table S2). The majority of these identified mRNAs (18,464) were expressed in both Jinhua and Yorkshire pigs, while 1,492 were Yorkshire-specific and 2,479 were Jinhua-specific. Most of the breed-specific mRNAs were expressed at low FPKM, suggesting these transcripts were generally expressed at low levels in the thyroid gland, being hard to discover. Tg mRNA exhibited the highest abundance in both pig breeds, accounting for 13.37% and 16.12% of all thyroid transcripts expression in Yorkshire and Jinhua pigs, respectively, which was consistent with previous studies [36]. TH-production-related transcripts (i.e., *HSPA5*, *IYD*, *DUOX2*, *SLC5A5*, *LRP2*, and *TSHR*) were also highly expressed in the pig thyroid, reaching more than 1000 FPKM. KEGG analysis showed that the transcripts whose expression levels were more than 1000 FPKM were enriched in the ribosome, focal adhesion, proteasome, fatty acid metabolism, spliceosome, and regulation of actin cytoskeleton pathways, suggesting that these mRNAs might be essential for maintaining the basic functions of the thyroid gland.

### 3.4. Identification of lncRNA

**Figure 1:**
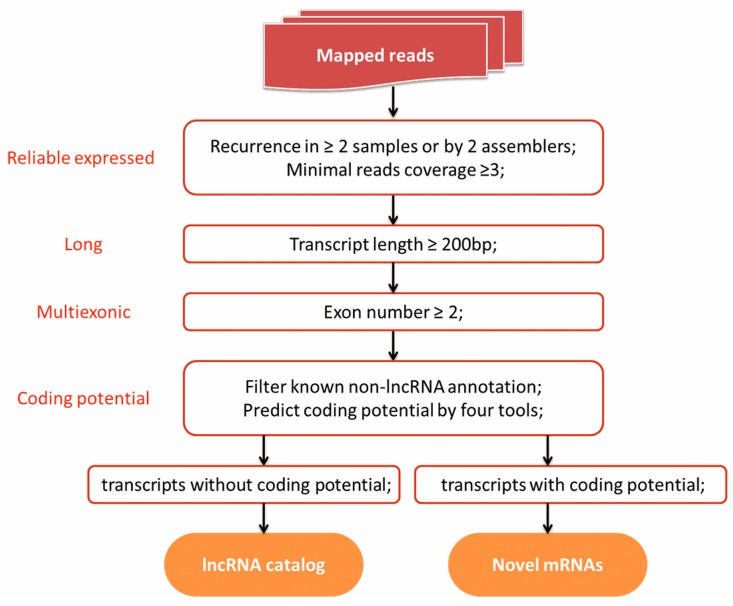
Schematic diagram of the pipeline used in the identification of long non-coding RNAs (lncRNAs) and novel mRNAs. This pipeline took the reads that mapped to the pig reference genome as input data (top). The four criteria required for the identification of lncRNAs are indicated in red. LncRNAs are defined as reliably expressed, long, multi-exonic, and noncoding transcripts. Transcripts that predicted with positive coding potential by any of the four tools (CNCI, CPC, Pfam-scan and PhyloCSF) were considered as novel mRNAs.

A series of filtering criteria was applied to define a set of putative lncRNAs in the porcine thyroid transcriptome (Figure 1). After assembling, 265,605 transcript isoforms were assembled by Cufflinks and Scripture. Putative lncRNAs were distinguished from the numerous lowly-expressed, single-exon, unreliable fragments, employing a read coverage threshold [12] and focusing only on multiexonic transcripts [21] with at least 200 bp. These procedures filtered 36,691 transcripts. Finally, annotated non-lncRNA transcripts (e.g., annotated protein-coding genes, pre-microRNA, tRNAs, rRNA, and pseudogenes) and transcripts with coding potential were eliminated, and a rigorous lncRNA set including 1018 transcripts was obtained (Table S3). Among these, 659 (64.7%) candidate lncRNA transcripts were included in the pig ALDB database (Table S4).

Blasting candidate lncRNAs to human and mouse lncRNA revealed 473 and 237 lncRNAs showed significant sequence similarity with human and mouse lncRNA, respectively (Table S5). Furthermore, 198 candidate lncRNAs showed sequence similarity with known lncRNAs in both human and mouse.

### 3.5. Identification of Novel mRNAs

Transcripts that predicted with coding potential by any of the four tools (CNCI, CPC, Pfam-scan, and PhyloCSF) were considered as novel mRNAs (Figure 1). We detected 1189 novel mRNAs not linked to any annotated genes in the NCBI database (Table S6).

### 3.6. Differential Expression Analysis

**Figure 2:**
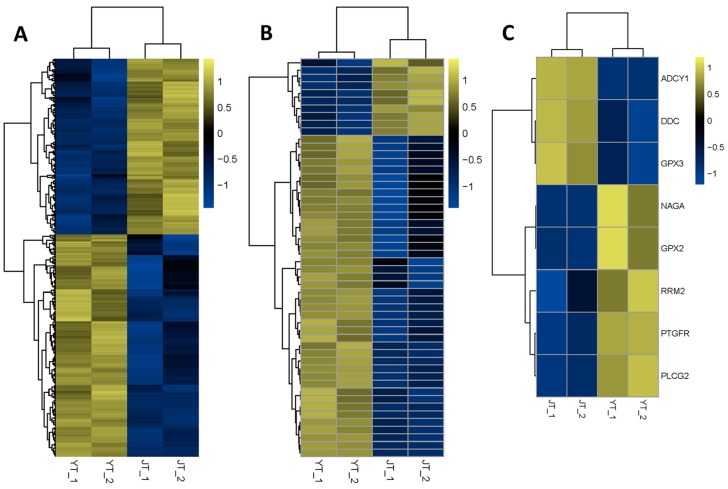
Heat maps of the distinguishable expression profiles in the thyroid gland of Jinhua and Yorkshire pig breeds. (**A**) Hierarchical clustering of the differentially-expressed mRNAs; (**B**) Differentially-expressed lncRNA transcripts; and (**C**) TH-regulation-related genes is performed. Yellow indicates relatively high expression and blue denotes relatively low expression.

Differentially-expressed mRNAs and lncRNAs in the thyroid glands of Yorkshire and Jinhua pigs were obtained by Cuffdiff. In Yorkshire pigs, 246 known mRNAs and 29 novel mRNAs were upregulated, whereas 209 known mRNAs and eight novel mRNAs were downregulated (*q*-value < 0.05) (Table S7). From the lncRNA expression data, 42 lncRNA transcripts were upregulated and 10 lncRNAs were down-regulated (*q*-value < 0.05) in the Yorkshire pigs (Table S8). The heat maps displayed mRNA (Figure 2A) and lncRNA (Figure 2B) significantly differentially-expressed between the two pig breeds.

Due to the importance of THs in pig growth and lipid metabolism, we focused on TH-regulation-related genes identified in previous studies (Figure 2C) [7]. The expression levels of transcripts in the calcium signaling pathway (*PTGFR*, *PLCG2*), lysosome (*NAGA*), tyrosine metabolism (*DDC*), and glutathione metabolism (*RRM2*) differed between Yorkshire pigs and Jinhua pigs. In addition, the expression of the TH-synthesis-related genes *ADCY1*, *GPX2*, and *GPX3* differed significantly between the two breeds.

### 3.7. Functional Enrichment of Differentially-Expressed mRNAs

The GO enrichment analysis performed to explore the functions of the differentially-expressed mRNAs between two pig breeds revealed that only the microtubule-based process (ontology: biological process) was significantly enriched (*p*-adjust < 0.05). In addition, the majority of mRNAs (10/14) assigned to this GO term were significantly upregulated in Yorkshire pigs (Table S9).

The KEGG pathway analysis performed to uncover biological functions of these genes indicated that the only significantly enriched network was “Cell cycle” (*p*-adjust < 0.05). Proliferating cell nuclear antigen (*PCNA*) was assigned to this pathway, and had a much higher expression level in Yorkshire pigs than Jinhua.

### 3.8. Functional Prediction of lncRNAs

**Figure 3:**
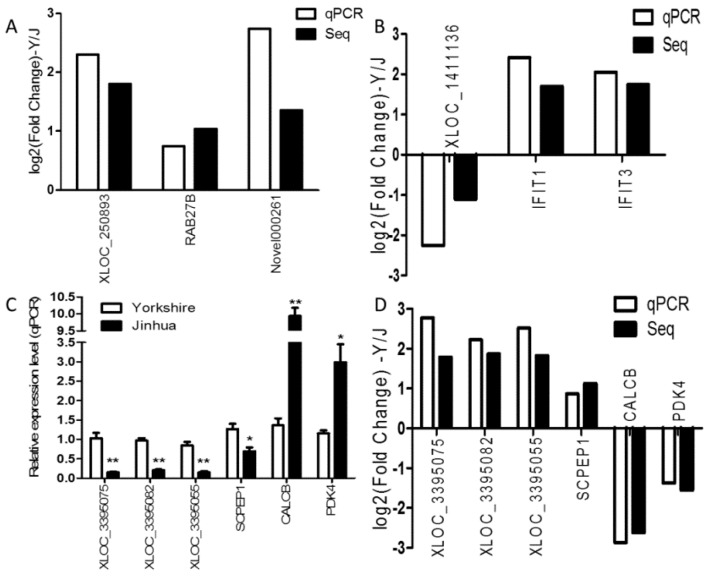
Validation of the RNA-seq data by qRT-PCR. (**A**) The expression patterns of *XLOC_250893* and its target genes (*RAB27B* and *Novel000261*); and (**B**) for *XLOC_1411136* and its target genes (*IFIT1* and *IFIT3*) were verified by qRT-PCR; (**C**) The relative expression levels of six randomly selected genes were expressed as the mean ± SEM. * *p* < 0.05, ** *p* < 0.01; (**D**) Fold-change of the Yorkshire pigs versus the Jinhua pigs were verified by qPCR.

To investigate the function of lncRNAs, we predicted the potential targets of lncRNAs in cis, using 100 kb as the cutoff. Thirteen differently expressed protein-coding genes were found close to 10 differentially-expressed lncRNA genes (Table S10). Seven of these 10 lncRNA genes were predicted to regulate only one target gene, while three were predicted to regulate two target genes. In summary, nine of the lncRNA-mRNA gene pairs were regulated in the same direction (up-up) and four pairs in the opposite direction (down-up). The expression levels of two target genes (*RAB27B* and *Novel000261*) for *XLOC_250893*, which were regulated in the same direction (Figure 3A), and of two targets (*IFIT1* and *IFIT3*) for *XLOC_1411136*, which were regulated in the opposite direction (Figure 3B), were validated by qRT-PCR.

### 3.9. Quantitative Real-Time PCR Validation

To validate RNA-seq results, three differentially-expressed mRNAs (*SCPEP1*, *CALCB*, and *PDK4*) and three differentially-expressed lncRNAs (*XLOC_3395075*, *XLOC_3395082*, and *XLOC_3395055*) were subjected to qRT-PCR, with three biological replicates for each breed (Figure 3C). As shown in Figure 3D, the relative fold changes in expression detected by qRT-PCR were consistent with RNA-seq data, suggesting our transcripts identification and abundance estimation were highly reliable.

### 3.10. Genomic Features of lncRNAs

**Figure 4:**
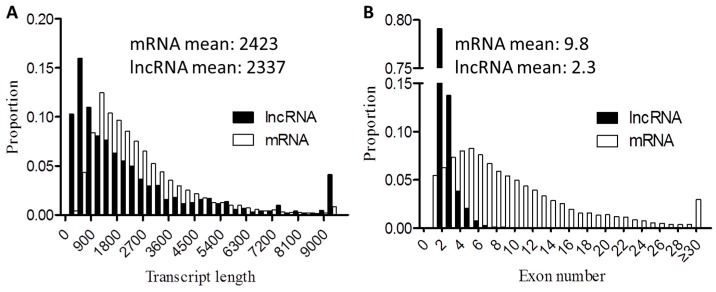
Genomic features of candidate lncRNAs. (**A**) Length distribution of the 38,222 coding transcripts (white) and 1,018 predicted lncRNAs (black); (**B**) Exon number distribution of coding transcripts and lncRNAs.

The candidate lncRNAs identified in this study were shorter in transcript length and contained fewer exons than mRNAs (Figure 4), which was consistent with previous studies [22,37].

## 4. Discussion

Although animal growth and fat deposition are complex processes regulated by many factors, the thyroid gland is one of the most important. It synthesizes, stores, and secretes THs, which are controlled by thousands of molecules, but until now, little is known about the porcine thyroid transcriptome. In this study, we conducted a preliminary investigation of mRNA and lncRNA expression profiles in the thyroid gland of Yorkshire and Jinhua pigs to assess the potential regulators of porcine growth and fat deposition related to thyroid functions.

Dramatic differences in growth performance and fat deposition were observed between Yorkshire and Jinhua pigs. Yorkshire pigs had significantly higher levels of TT3 and TT4 than Jinhua pigs. Serum levels of FT4 and FT3 in the Yorkshire pig also tended to be higher than in the Jinhua pig. These results are in agreement with those of a previous study reporting that serum FT3 and FT4 levels in Jinhua pigs were significantly lower than those in Landrace pigs, which is another lean-type pig breed [38].

To our knowledge, this is the first application of Ribo-zero RNA sequencing to study the porcine thyroid transcriptome. This method allows non-polyA transcripts to be obtained, as well as fragmented RNAs, which is advantageous compared to polyA sequencing [39]. Most of the 351 million reads obtained (83%) were mapped to the current *S. scrofa* genome assembly, which was higher than the percentage obtained for testis (77.4%–78.37%) [40] and muscle (79.31%–82.21%) transcriptomes [41]. The extensive sequencing depth yielded high sequence read coverage on the pig reference genome. With the higher sequencing depth and higher mapping ratio, we obtained more transcripts. However, a large proportion of clean reads (33%–39%) were mapped to unannotated loci, reinforcing the need to improve current pig genome annotation.

A total of 22,435 known and 1189 novel mRNAs were expressed in this study, and almost all novel mRNAs had low expression, which suggested that their roles in the thyroid need to be further confirmed. The 492 differentially-expressed mRNAs between Jinhua and Yorkshire pigs might explain the different levels of THs found between the two pig breeds, as many important differentially-expressed genes participate in TH synthesis and secretion. It is well established that glutathione metabolism, lysosome, tyrosine metabolism, and calcium signaling pathway are involved in the synthesis and secretion of THs [7], and any change in these pathways might affect the levels of serum THs. The present study results evidenced differentially-expressed genes in all these pathways. More importantly, the majority of the differentially-expressed genes assigned to these pathways were significantly upregulated in Yorkshire pigs, which might improve the effect of the upstream regulator on TH production. Notably, our study also showed that the expression of TH-synthesis-related genes was significantly different between Yorkshire and Jinhua pigs. For example, adenylate cyclase 1 (*ADCY1*) is activated by *TSHR* and involved in the process of adopting and organifying iodide [42]. Glutathione peroxidase 2 (*GPX2*) and Glutathione peroxidase 3 (*GPX3*)—which are induced by TSH in vitro [43]—participate in the degradation of thyroglobulin and in the liberation of T4 and T3 [44,45]. Anoctamin 1 (*ANO1*) is a calcium-activated anion channel with a preferential affinity for iodide over chloride, and may contribute to the rapid delivery of iodide into the follicular lumen for the synthesis of THs [46,47], and improve the TH-secretion response to extracellular stimulus (such as TSH, iodine). Secretogranin III (*SCG3*) was also reported as upregulated in thyroid regeneration [48]. In addition, a group of differentially-expressed genes were reported to be related to thyroid diseases, such as caspase 8 (*CASP8*) [49], dual specificity phosphatase 4 (*DUSP4*) [50], BTG family, member 2 (*BTG2*) [51], potassium channel, voltage gated Shal related subfamily D, member 3 (*KCND3*) [52], interferon-induced protein with tetratricopeptide repeats 1 (*IFIT1*) [53], chloride channel, voltage-sensitive 5 (*CLC-5*) [54] and connective tissue growth factor (*CCN2*) [55]. In this study, 14 genes assigned to the GO term "microtubule-based process" were differentially regulated between the two pig breeds, and previous studies suggested a role for microtubules in thyroid secretion through endocytosis [56,57]. The currently accepted mechanism for TH secretion is that TG molecules are first taken up by polarized thyrocytes through vesicle-mediated endocytosis, and then transported to the lysosome for proteolytic cleavage, releasing T4 and T3. Thus, the first step is the limiting point in the TH secretory pathway. Taken together, differential expression of these genes may lead to a difference in thyroid function and serum THs concentration, which may partly explain the different growth rate and fat deposition between Yorkshire and Jinhua pigs, although their exact mechanism needs further investigation.

Given their low expression level and few annotated functions, lncRNAs were thought to be transcriptional noise. With the development of high-throughput sequencing technologies for large-scale expression profile analysis, the discovery and characterization of lncRNAs became feasible [58,59]. A total of 1018 candidate lncRNA transcripts were identified in this study. This number is much lower than the pig lncRNAs in the ALDB database (12,103) [60]. One possible reason for this difference is that lncRNAs are expressed in a tissue-specific pattern [61], and many of the lncRNAs in the ALDB are exclusively expressed in other tissues. This also partly explains why only 64.7% of the candidate lncRNAs identified in this study were included in the ALDB database. Similar to the lncRNAs in other species [31,62], the candidate lncRNAs identified in pig thyroid are shorter, have fewer exons, and lower expression levels than mRNAs.

Analyzing conservation of lncRNAs across species might help understand lncRNA evolution and function [63]. The comparative analysis found that 473 (46.5%) and 237 (23.3%) candidate lncRNAs showed significant sequence similarities with human and mouse lncRNAs, respectively. Furthermore, 198 (19.4%) lncRNAs showed significant sequence similarities with both human and mouse lncRNAs. These observations are consistent with the results of Zhou et al., who reported a 40% similarity between pig and human long intergenic non-coding RNAs [64], suggesting that pig might be a good model to analyze lncRNAs function. Compared to the more than 84% sequence similarity between pig and human mRNAs [65], the homology between pig and human lncRNAs is small, supporting the hypothesis that lncRNA evolved more rapidly than mRNA [31,63].

Fifty-two lncRNA transcripts were significantly differentially expressed in the thyroid gland of Yorkshire and Jinhua pigs, and they might play critical roles in porcine thyroid function. The roles of some lncRNAs in the development and function of some endocrine organs (pancreatic β cells, white and brown adipose tissue, adrenal gland, and mammary gland) have been reported in previous studies [13]. The function of lncRNAs was not inferred from their sequences and structures. In addition, recent studies have demonstrated that some lncRNAs can regulate the expression levels of their chromosomal neighboring genes [66], and this has been used to predict the function of lncRNAs [12,62,67]. In the present study, we searched for potential target genes 100 kb up- and downstream of lncRNAs. To minimize the false positive rates to predict the target genes of lncRNAs, only differentially-expressed lncRNAs and mRNAs were used in this analysis, which found 13 potential lncRNA-mRNA gene pairs. These were either co- or inversely-regulated, in agreement with what has been reported in previous studies [68,69]. Co-regulation of lncRNA-mRNA pairs suggested they might be under the same transcriptional control or that lncRNAs activated the expression of their target genes. Ørom et al. reported some lncRNAs display an enhancer-like function on their neighboring mRNAs [70]. Inverse expression patterns between lncRNAs and their neighboring protein-coding genes indicate that lncRNAs might silence or inhibit the expression of their target genes. For example, the *Xist* lncRNA is expressed from one of the two X chromosomes in female mammals, and induces silencing of the whole chromosome [71]. We also found that some lncRNAs might exert their function in the pig thyroid through their potential target mRNAs. For example, lncRNA *XLOC_250893* was predicted to act on *Rab27b*, a member of the Rab family of small GTPase proteins. Rab GTPases regulate almost all membrane trafficking processes, including vesicle formation, movement, and fusion [72]. *Rab27b* has been reported to be widely expressed in secretory organs such as the pituitary gland, pancreas, and thyroid gland [73], and is up-regulated in papillary thyroid carcinoma [74]. LncRNA *XLOC_1411136* was predicted to act on the potential garget gene *IFIT1*. Kuang et al. reported that *IFIT1* might play an important role in the pathology of Graves’ disease [53], an autoimmune disorder of the thyroid gland characterized by hyperthyroidism. These findings suggest that lncRNAs might play regulatory roles in pig thyroid function and health. However, these predicted lncRNA-mRNA pairs require further experimental verification.

## 5. Conclusions

This study represented the first description of mRNA and lncRNA profiles in the porcine thyroid employing Ribo-Zero RNA-seq technology. In addition, the 1189 novel mRNAs and 1018 candidate lncRNA transcripts detected, 22,435 known mRNAs were expressed in the porcine thyroid. Among the 455 known mRNAs, 37 novel mRNAs, and 52 lncRNA transcripts differentially-expressed in Yorkshire and Jinhua pigs, several were known to regulate the synthesis and secretion of THs, according to previous studies. Bioinformatics analysis suggested that differentially-expressed lncRNAs might also play a role in regulating the synthesis and secretion of THs by governing their potential target genes. Considering the known important role of THs in metabolism, the mRNAs and lncRNAs revealed in this study would contribute to the understanding of the possible mechanisms involved in animal growth and fat deposition, although more detailed studies are required.

## Figures and Tables

**Table 1:** Body Index and serum concentration of thyroid hormones (THs) in the Jinhua and Yorkshire pig breeds.

Traits	Jinhua	Yorkshire	*p*-Value
Body weight (kg)	29.33 ± 0.88	47.07 ± 1.93	0.001
Thyroid gland weight (g)	3.20 ± 0.06	4.93 ± 0.38	0.011
Thyroid index (g/kg)	0.109 ± 0.004	0.105 ± 0.009	0.702
Serum total thyroxine level (nmol/L)	30.36 ± 0.76	37.85 ± 1.73	0.017
Serum total triiodothyronine level (nmol/L)	0.53 ± 0.02	1.00 ± 0.12	0.020
Serum free thyroxine level (pmol/L)	9.34 ± 0.20	11.31 ± 0.73	0.058
Serum free triiodothyronine level (pmol/L)	1.84 ± 0.09	2.58 ± 0.33	0.096

All data are expressed as mean ± SEM; and a *p*-value < 0.05 reveals significant differences.

**Table 2:** Summary of the sequencing reads alignment to the reference genome.

Sample	Total Reads	Total Mapped %	Uniquely Mapped %
Jinhua-1	89460072	81.83	70.63
Jinhua-2	90102440	81.50	72.83
Yorkshire-1	84160098	83.63	72.62
Yorkshire-2	87285462	84.37	73.80

## Supplementary Materials

The following are available online at http://www.mdpi.com/2073-4425/7/7/34/s1, Table S1: Categorization of reads annotation, Table S2: Known mRNAs identified in porcine thyroid, Table S3: Candidate lncRNA identified in porcine thyroid, Table S4: Candidate lncRNAs included in pig ALDB database, Table S5: Candidate lncRNAs with sequence similarities with human or mouse, Table S6: Novel mRNA identified in porcine thyroid, Table S7: Differentially-expressed mRNAs, Table S8: Differentially-expressed lncRNAs, Table S9: Genes involved in microtubule-based process, Table S10: LncRNA-mRNA pairs, Table S11: Primers for qRT-PCR.

## Abbreviations

The following abbreviations are used in this manuscript:

T3	Triiodothyronine
T4	Tetraiodothyronine or Thyroxine
THs	Thyroid Hormones
PCR	Polymerase Chain Reaction
FPKM	Fragments per kilo-base of exon per million mapped fragments
